# Valproic Acid: Protective or Triggering in Paranoid Psychotic Symptoms? — A Case Report After Long-Term Valproic Acid Discontinuation

**DOI:** 10.1192/j.eurpsy.2026.12192

**Published:** 2026-07-31

**Authors:** T. Başyiğit, I. Keçeci, E. Çiftçi

**Affiliations:** NP İstanbul Beyin Hastanesi, İstanbul, Türkiye

## Abstract

**Introduction:**

Valproic acid (VPA) is a widely preferred and frequently prescribed antiepileptic drug used in the treatment of both generalized and focal epilepsies worldwide. Due to its broad-spectrum efficacy and generally good tolerability, it holds an important place among standard treatment options. However, although rare, serious adverse effects may occur in some patients, including hemorrhagic pancreatitis, coagulation disorders, bone marrow suppression, VPA-induced hepatotoxicity, and encephalopathy. Additionally, very rare but clinically significant neurological adverse effects have been reported, such as VPA’s so-called *paradoxical effect*, characterized by increased seizure frequency without VPA-induced encephalopathy, and brain pseudoatrophy. The incidence and mechanisms underlying these specific adverse effects remain unclear in the literature. Moreover, treatment outcomes and definitive indications regarding VPA use have not yet been fully established.

**Objectives:**

To evaluate the dissociative symptoms and paranoid psychotic features that emerged during and/or after long-term VPA treatment discontinuation, and to discuss the potential effects of VPA on these psychiatric manifestations.

**Methods:**

A retrospective case report. Medical records, psychiatric assessments, MRI, and EEG findings were reviewed.

**Results:**

A 48-year-old female was started on VPA 1000 mg/day at 13 after an epileptic seizure and continued for 25 years. At 39, epilepsy was ruled out, and VPA discontinued. About a year later, after an emotional stressor, she developed depressive mood, paranoid delusions, and psychotic features. Partial improvement occurred with lithium and risperidone, but she discontinued them due to side effects. Untreated periods worsened psychotic symptoms, including persecutory delusions and surveillance thoughts. Trials of amisulpride and aripiprazole caused akathisia and parkinsonian symptoms, leading to non-adherence. During the drug-free period, delusions severely impaired functionality, and lithium and risperidone were given. Tardive dyskinesia emerged, for which bornaprine was started. While on bornaprine, lithium, and risperidone, she developed dissociative symptoms, prompting a switch to lithium, valproic acid, clozapine, fluvoxamine, and bornaprine. After reintroducing VPA, marked improvement in psychotic and dissociative symptoms occurred.

**Conclusions:**

This case suggests that VPA may exert a stabilizing and possibly protective effect on mood and psychotic symptoms in vulnerable individuals. Discontinuation of long-term VPA treatment may contribute to the emergence or worsening of paranoid psychosis. Psychiatric monitoring is advised after VPA withdrawal, especially in patients with affective dysregulation or psychotic predisposition. Further studies are required to clarify whether VPA functions as a protective factor against psychosis.

**Disclosure of Interest:**

None Declared

